# Autoantibodies against the Immunoglobulin-Binding Region of Ro52 Link its Autoantigenicity with Pathogen Neutralization

**DOI:** 10.1038/s41598-018-21522-7

**Published:** 2018-02-20

**Authors:** Peter D. Burbelo, Leyla Y. Teos, Jesse L. Herche, Michael J. Iadarola, Ilias Alevizos

**Affiliations:** 1 Dental Clinical Research Core, National Institute of Dental and Craniofacial Research, National Institutes of Health, Bethesda, MD 20892 USA; 2 Sjögren’s Syndrome and Salivary Gland Dysfunction Unit, National Institute of Dental and Craniofacial Research, National Institutes of Health, Bethesda, MD 20892 USA; 3 Secretory Mechanisms and Dysfunction Section, National Institute of Dental and Craniofacial Research, National Institutes of Health, Bethesda, MD 20892 USA; 4 Department of Perioperative Medicine, Clinical Center, National Institutes of Health, Bethesda, MD USA

## Abstract

Ro52/TRIM21 plays a key role in antibody-dependent pathogen neutralization and is a major autoantigen in systemic lupus erythematosus, Sjögren’s syndrome (SS), and other autoimmune diseases. Here we evaluated immunoreactivity against Ro52-related molecules in SS and healthy volunteers. Although most proteins examined were not antigenic, several TRIM paralogs, including TRIM22, and TRIM38, showed sporadic immunoreactivity in SS. In contrast, the murine Ro52 ortholog with limited linear homology demonstrated high levels of autoantibodies implicating the importance of shared conformational epitopes. To further explore the autoantigencity of Ro52, deletion and point mutant analyses were employed revealing previously hidden, robust autoantibodies directed against its C-terminal immunoglobulin-binding domain. Another autoantibody, rheumatoid factor, targeting the Fc region of IgG, strongly overlapped with Ro52 seropositivity (odds ratio 14; *P* < 0.0001). These convergent mechanistic findings support a model whereby intracellular Ro52-bound antibody-coated pathogen complexes, released or misprocessed from infected cells, drive autoantigenicity against Ro52 and the Fc region of IgG.

## Introduction

SSA autoantibodies, originally discovered in the 1960s^1^, are important clinical biomarkers for the diagnosis of a number of autoimmune diseases including myositis, scleroderma, systemic lupus erythematosus, and Sjögren’s syndrome^2^. Molecular characterization of the SSA autoantigen revealed it was composed of two proteins, Ro52/TRIM21 (hereafter referred to as Ro52)^3^ and Ro60^4,5^. Although Ro52 and Ro60 are structurally and functionally unrelated, the levels of autoantibodies directed against these two autoantigens often track each other^2^. Understanding the mechanisms of antigenicity of Ro52 and Ro60 may shed light on the pathogenesis of systemic lupus erythematosus, SS and other autoimmune diseases.

Ro52 is a member of a large group of over 60 human TRIM proteins key for signal transduction and innate immunity^6^. Although Ro52 is ubiquitously expressed, it is enriched in immune cells^7–9^. Ro52 contains an N-terminal ubiquitin ligase domain and C-terminal PRYSPRY domain^10^ and interacts with and ubiquitinates a variety of molecules involved in different inflammatory and signal transduction pathways including IRF8^11^, IRF3^12^, IKK-beta^13^, GMPS^14^, NMI^15^, and SQSTM1^16^. Besides controlling these different signaling pathways, multiple seminal studies by James and colleagues have recently found that Ro52 acts as an immunoglobulin-binding receptor and functions in antibody-dependent intracellular neutralization (ADIN) of pathogens^17–25^. The first evidence for this activity came from a yeast two-hybrid screen showing Ro52 directly interacts with immunoglobulin heavy chains^26^. Subsequent interaction mapping demonstrated that the C-terminus of Ro52 is necessary and sufficient for immunoglobulin binding activity and does not require the N-terminal ubiquitin ligase domain^27^. X-ray crystallography of the C-terminus of the Ro52 protein revealed the structural basis of the domain’s interactions with immunoglobulins and identified key contact amino acid residues^28^. Importantly, *in vitro* and *in vivo* functional studies have shown that Ro52 binds intracellular antibody-bound pathogens including non-enveloped viruses such as adenovirus^22,24^ and *Salmonella enterica* bacteria^21^, leading to their destruction by the ADIN pathway^17^. During this process, the intracellular binding of Ro52 to antibody-coated pathogens triggers E2 UbeW monoubiquitination of Ro52, which subsequently drives destruction of the pathogen complex in the proteasome^18–20^. Additional recruitment of other E2 Ube2N/Ube2V2 ubiquitin E2 enzymes causes conjugation of lysine 63-linked ubiquitin to Ro52 and is followed by subsequent cleavage of ubiquitin and release leading to signal transduction activation of the cGAS and Rig-I pathways^23^. These and other findings highlight Ro52’s critical role in the intracellular clearance of antibody-bound pathogens.

Although Ro52 has been extensively examined in the context of cell signaling and host defense, little is known about why it is an autoantigen in certain autoimmune diseases. One limitation of many clinical studies has been the analysis of autoantibodies against the SSA complex, containing both Ro60 and Ro52, rather than Ro52 by itself^10^. In addition, immunoassays examining Ro52 immunoreactivity have almost all involved solid phase immunoassay formats, such as ELISA and peptide arrays, which poorly detect conformational epitopes. Studies published to date employing these technologies have only detected autoantibodies directed against the N-terminus of Ro52^29,30^, including a leucine zipper region as a key antigenic region^29–31^. Compared to solid-phase immunoassays, fluid-phase immunoassays, such as luciferase immunoprecipitation systems (LIPS), are much more sensitive^32^. Particularly relevant for SS is the findings by two independent groups that the LIPS assay detected La autoantibodies in SS with approximately 60% sensitivity (100% specificity) compared to 45% by ELISA^33,34^. Moreover, we have demonstrated high levels of Ro52 autoantibodies particularly against the N-terminal half of the molecule in SS^33,35,36^ and systemic lupus erythematosus^37^. In the current study, we used the LIPS technology to examine potential mechanisms of autoantigenicity of Ro52 by exploring functionally regulated pathways, structurally related molecules and immunoreactivity within the C-terminus of Ro52.

## Results

### Ro52 co-regulated genes as potential autoantigens

**Figure 1:**
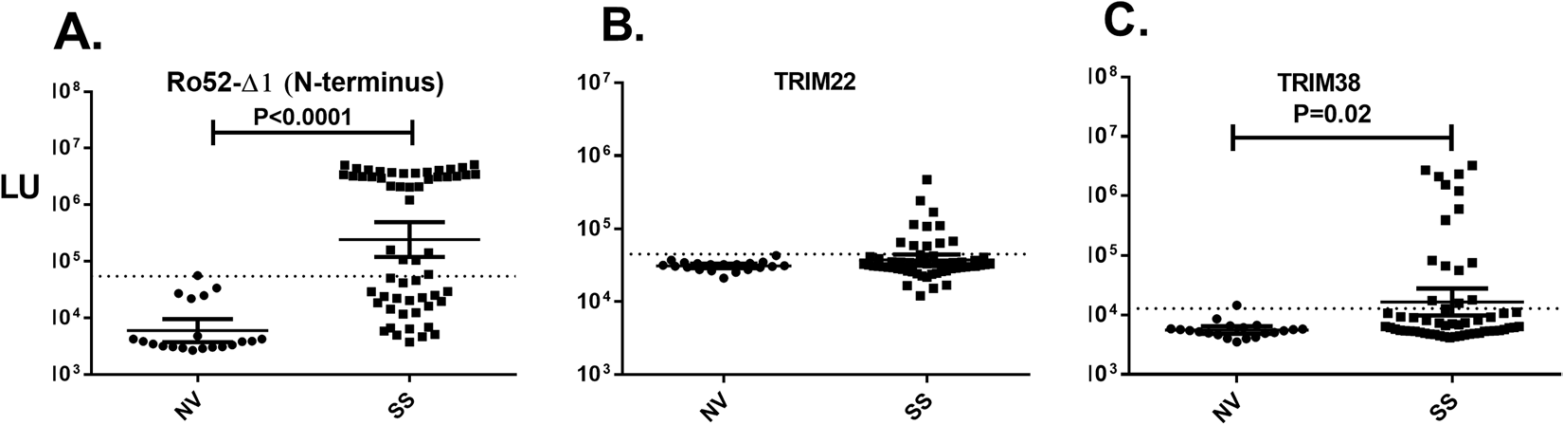
Detection of autoantibody against Ro52, TRIM22 and TRIM38 in SS. Scatter plot graphs represent autoantibody levels determined by LIPS in individual subjects from a cohort of 20 normal volunteers (NV) and 57 subjects with SS. The luminescence of autoantibody response (LU) for each subject are plotted on the Y-axis. (**A**) Autoantibodies against Ro52 were determined using a previously described N-terminal protein fragment (Ro52-Δ1). Autoantibodies against (**B**) TRIM22 and (**C**) TRIM38, two potential Ro52 co-regulated genes, are also shown. For each plot, the solid line represents the geometric mean plus the 95% CI. For each antigen, a cutoff value shown by the dotted line was based on the mean plus three SD of the autoantibody values observed in normal volunteers. *P* values were calculated using the Mann-Whitney *U* test and only P values < 0.05 are shown.

Sjögren’s syndrome and other autoimmune diseases display high levels of autoantibodies against Ro52. Here, we explored the possibility of Ro52 co-regulated gene products as autoantigens. This approach is based in part on studies showing that genes co-regulated at the transcriptional level are often present in similar protein complexes and signaling pathways^38^. To identify Ro52-coregulated genes, we mined an available database called SEEK (search-based exploration of expression compendia; SEEK@princeton.edu/), which provides datasets of co-regulated genes based on transcriptomic data^39^. Analysis of the 30 most co-regulated genes revealed several genes encoding proteins involved in the proteasome, immune signaling as well as two structurally-related TRIM proteins **(**Supplemental Table 1). To determine if any of the co-regulated gene products were autoantibody targets, we generated ten proteins representative from these pathways, including PSMB9, PSMB8, PSME1, IRF-1, SAMD9L, DTX3L, IFI35, NMI, TRIM38, and TRIM22, as light-emitting fusion proteins for LIPS autoantibody testing. We then used these recombinant proteins to evaluate healthy volunteers (n = 20) and fifty subjects with SS (n = 57). Analysis of the autoantibody profile against an N-terminal protein (Ro52-Δ1) fragment known to exhibit high performance for the diagnosis of SS^33,35^ revealed a wide dynamic range in Ro52 autoantibody levels in the cohort ranging from 2000 to 5 million light units (LU) (Fig. 1A). The geometric mean level (GML) of Ro52 autoantibodies in the SS subjects was 244,290 LU (95% CI: 120,100–496,700) and was 41 times higher than the GML of 5980 LU (95% CI: 3,743–9,555) in the healthy volunteers. Using a cut-off value derived from mean plus three standard deviations of the 20 heathy controls, revealed that the Ro52 autoantibody test had 59% sensitivity and 100% specificity in the diagnosis of SS (Fig. 1B). In contrast, many of the candidate co-regulated molecules, including PSMB9, PSMB8, PSME1, IRF1, SAMD9L, DTX3L, IFI35, and NMI, exhibited no significant autoantigenicity (i.e. less than 1% sensitivity) in the SS subjects compared to the healthy volunteers (data not shown). However, two TRIM proteins, TRIM22 **(**Fig. 1B) and TRIM38 (Fig. 1C) demonstrated immunoreactivity in a sub-population of SS subjects. TRIM22 autoantibodies were present in 18% of the SS subjects (100% specificity), but this was not statistically significant (Fisher’s exact test; p = 0.057). TRIM38 autoantibodies were detected in 26% of the SS (15/57), which was statistically significant (Fisher’s exact test; p < 0.008) compared to their absence in the healthy blood donors (0/20). These findings suggest the possibility that these and other TRIM molecules might be minor autoantigens in SS.

### Ro52 is the primary TRIM autoantigen in SS

**Figure 2:**
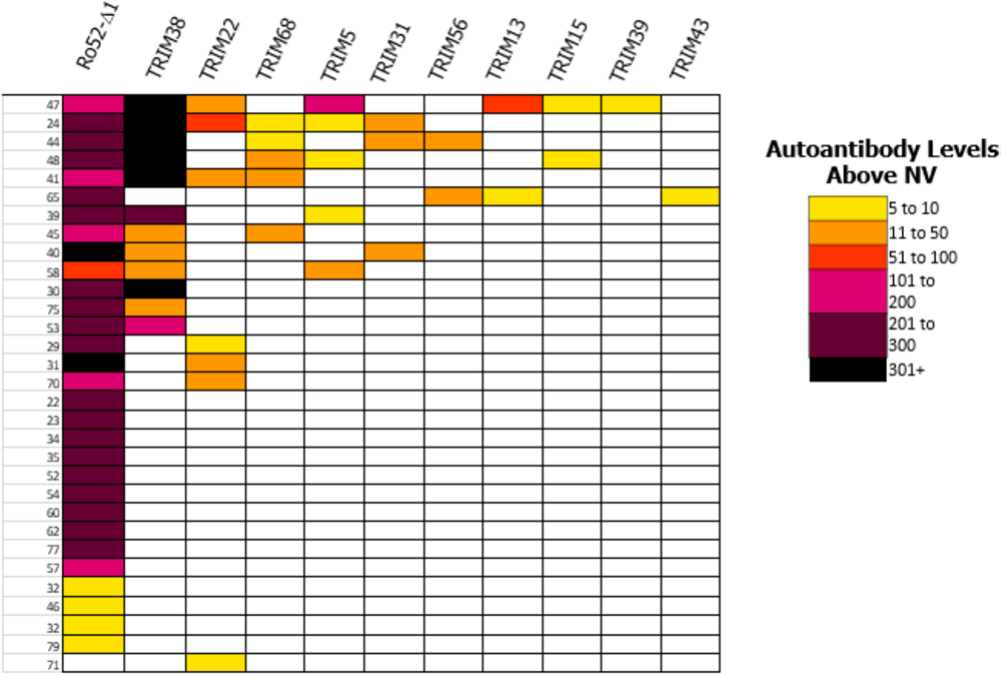
Heatmap analysis of autoantibodies against Ro52 and TRIM proteins in SS. Heatmap analysis of autoantibody responses against Ro52 and a panel of ten TRIM proteins in SS subjects are shown. Each row represents a single SS subject’s autoantibody profile. Clear boxes represent seronegative samples that matched the normal volunteer profile. Color coding shows antibody levels, expressed as integer multiples compared to the normal volunteer baseline. As denoted in the figure legend, these values in the SS subjects ranged from yellow (*Z* score from 5–10) to black (*Z* score < 301) reflecting extremely high autoantibody levels.

Due to the autoantibody immunoreactivity observed in SS against TRIM38 and TRIM22, we further examined eight additional structurally-related TRIM proteins as potential autoantigens, which were selected based on relevance to immune signaling and/or their sequence homology with Ro52. These TRIM proteins were roughly the same size as Ro52 and showed approximately 39–45% identity and 60–65% similarity at the amino acid level. LIPS autoantibody testing of the eight additional TRIM proteins as recombinant luciferase protein fusions revealed sporadic immunoreactivity in selected SS patients compared to the healthy volunteers, but the changes in prevalence was not statistically significant by Fischer’s exact testing (Supplemental Fig. 1). Some of the most immunoreactive TRIM proteins, TRIM5, TRIM56, TRIM43 and TRIM68, showed seropositive autoantibodies in 16%, 14%, 12% and 8% of SS subjects, respectively. To yield an overview of the TRIM protein immunoreactivity in SS, heatmap analysis was used to compare the Ro52 immunoreactivity profile with the ten TRIM proteins in the SS cohort (Fig. 2). As shown, Ro52 had the highest prevalence of seropositive autoantibodies in the SS cohort. TRIM38 had the next highest frequency of seropositivity, with about a third of the subjects showing very high autoantibody values. Notably, all the SS subjects with TRIM38 seropositivity were also Ro52 co-positive. TRIM22 was immunoreactive with seven of the samples including a low positive with one Ro52 seronegative SS subject (i.e. sample 71). Although the other eight TRIM molecules showed occasional immunoreactivity, these seropositive SS samples all overlapped subjects that were co-positive for Ro52 and TRIM38 (Fig. 2). The addition of the results for the other TRIM proteins did not improve the diagnostic performance of the existing Ro52 autoantibody test.

Based on the possibility that TRIM38 might be a minor autoantigen in SS, additional competition experiments were used to characterize the autoantibodies seen in the Ro52-TRIM38 co-positive subjects. In these experiments, unlabeled Ro52 was used as the competitor (Supplemental Table 2**)**. As shown, preincubation of six different co-positive patient sera with Ro52 recombinant protein, but not with control extract, efficiently blocked (i.e. 0–18% remaining) the autoantibodies directed against luciferase-Ro52 fusion protein in the LIPS assay. Under the same conditions, however, recombinant Ro52 only partially competed away (i.e. 28–54% remaining) of the autoantibody signal directed against TRIM38 (Supplemental Table 2). Together these results suggest that the immunoreactivity seen against TRIM38 involves both autoantibodies specific for TRIM38, as well as autoantibodies directed against shared epitopes of Ro52.

### Murine Ro52 efficiently detects human SS autoantibodies

**Figure 3:**
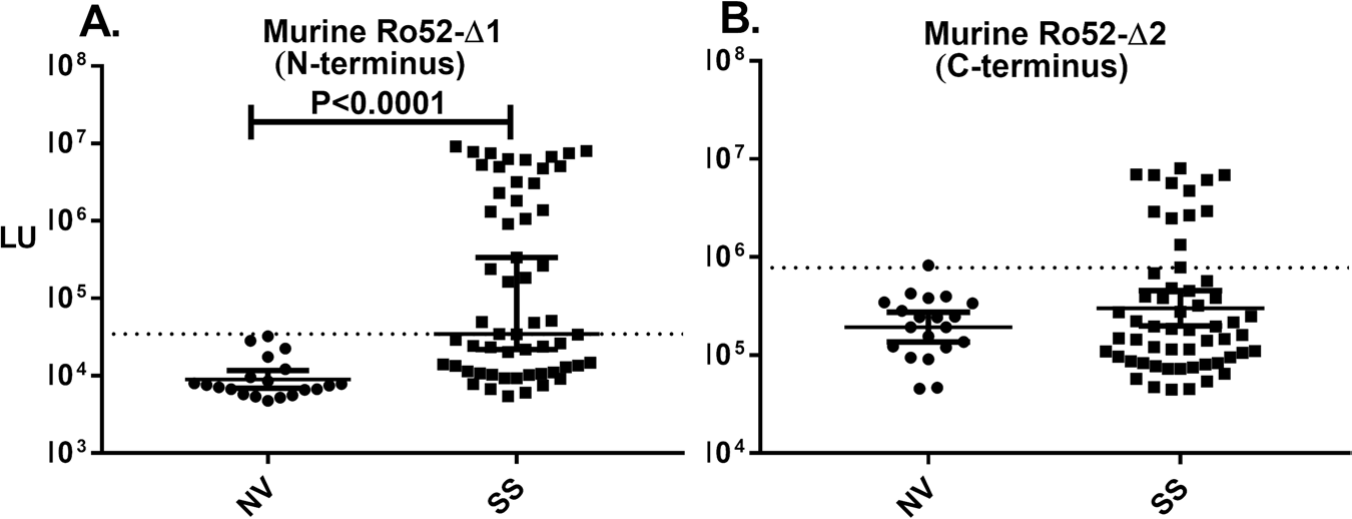
Detection of SS autoantibodies using murine Ro52 protein fragments. Autoantibodies were studied in the SS cohort using recombinant murine Ro52 proteins encompassing the (**A**) N-terminal and (**B**) C-terminal protein fragments. Cut-off values for each target were based on the antibody value obtained from the mean plus three SD for normal volunteers and is delineated by the dotted line. *P* values comparing the normal volunteers (NV) and SS subjects were calculated using the Mann-Whitney *U* test.

To further analyze the contribution of linear and conformational epitopes associated with Ro52, autoantibody responses against the murine version of Ro52 was studied. Sequence analysis of the murine Ro52 protein with the human version revealed 83% similarity and 70% identity. Despite this homology, few regions between both proteins showed more than eight amino acids of continuous alignment (Supplemental Fig. 2). LIPS testing of the murine N-terminal protein fragment (mRo52-Δ1) demonstrated autoantibodies in 28 of the 57 of the SS subjects, which was statistically significant (Fisher’s exact test; p < 0.0001) compared to their absence in the healthy blood donors (0/20). This diagnostic performance of 49% sensitivity and 100% specificity for detection of autoantibodies in the SS subjects was only slightly less informative than the corresponding human fragment (compare Fig. 3A with Fig. 1A). Robust autoantibody levels were detected in many of the SS subjects supporting the notion that autoantibodies are directed against shared Ro52 epitopes, which are retained even when stretches of identical amino acids are low. Moreover, these results are consistent with the hypothesis that the sporadic autoantibodies observed against the previously tested, related TRIM proteins likely represent shared epitopes with Ro52.

Additional analysis of autoantibodies against the murine C-terminal fragment (mRo52-Δ2) showed a high background in the LIPS assay, similar to our published study with the human C-terminal Ro52 fragment^35^. However as shown in Fig. 3B, this murine C-terminal Ro52 fragment more clearly identified autoantibodies than reported for the human ortholog and detected autoantibodies in 13 of the 57 of the SS subjects (23% sensitivity), which was statistically significant (Fisher’s exact test; p < 0.017). These results suggest that the C-terminus of murine Ro52 is a target of human SS autoantibodies.

### The immunoglobulin-binding domain of Ro52 is a target of autoantibodies

**Figure 4:**
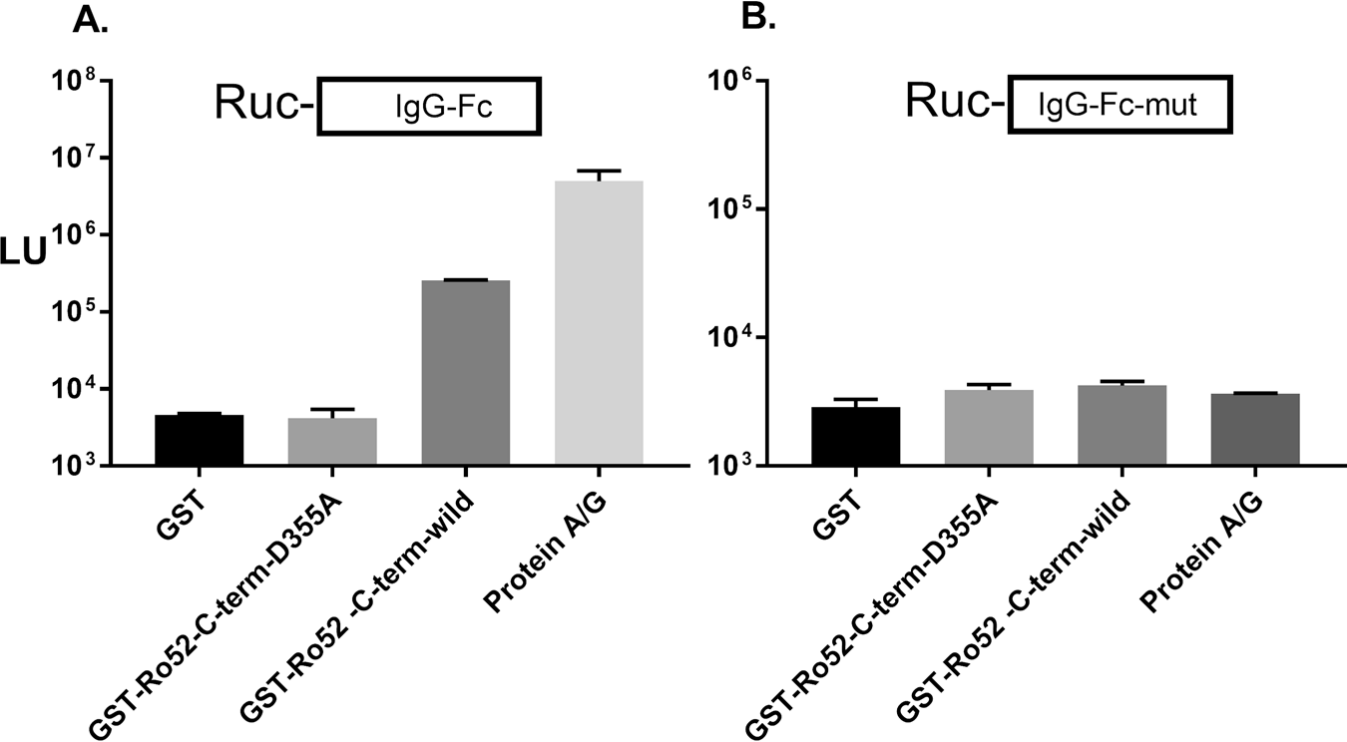
The C-terminus of Ro52 interacts with the Fc region of IgG1. Protein-protein interactions were detected using bead immobilized target proteins and a luciferase-tagged Fc-IgG recombinant protein. GST fusion proteins of wild type (Ro52-Δ2) and C-terminal mutant (Ro52-Δ2-D355A) Ro52 were tested for their ability to interact with (**A**) *Renilla* luciferase-IgG-Fc and (**B**) *Renilla* luciferase-IgG-Fc mutant. Following incubation, the beads were washed and then measured for LU. The mean from two experiments is shown along with the standard error. As a positive control, protein A/G beads were found to strongly bind IgG-Fc, but not the IgG-Fc mutant recombinant protein.

Amongst TRIM proteins, Ro52 is the only member known to have immunoglobulin-binding activity^40,41^. Given this property, we investigated the *in vitro* immunoglobulin binding capacity of Ro52’s C-terminus and developed a mutant protein deficient in this activity for further study of its autoantigenicity. To measure immunoglobulin binding activity, we employed a simple pull-down assay^42^ utilizing recombinant protein fragments of wild type human IgG1 Fc and mutant IgG1 Fc fused to *Renilla* luciferase. Using this assay, the known high affinity protein binding was first confirmed between the Fc portion of IgG and protein A and G^43,44^ showing that cell extract containing luciferase-IgG1-Fc recombinant protein, but not luciferase–IgG-mutant bound the protein A/G beads extremely well yielding high levels of LU (Fig. 4A). To study the interaction between Ro52 and the IgG1 Fc region, we used Cos-cell produced recombinant glutathione S-transferase (GST) protein fragments of the wild type C-terminal region of Ro52 and a corresponding mutant (Ro52-Δ2-D335A), potentially defective in immunoglobulin binding activity. We generated this mutant based on known crystal structure information of the C-terminus of Ro52 and previous functional binding data on mutants within this region^28^. The GST-recombinant wild type and mutant Ro52 protein fragments along with glutathione beads were then used in place of the protein A/G beads to measure the ability to bind and capture the luciferase tagged IgG1-Fc protein complexes. As shown in Fig. 4A, the luciferase-IgG Fc wild type protein showed very high binding to the glutathione beads bound to GST-Ro52-Δ2, but not with the glutathione beads bound to the GST-Ro52-Δ2-D335A mutant or glutathione beads by themselves. In contrast, the mutant IgG1-Fc protein showed little binding to glutathione beads, glutathione beads bound to GST-Ro52-Δ2, or glutathione beads bound to the GST–Ro52-Δ2-D335A mutant (Fig. 4B). These results confirm that the C-terminus of Ro52 contains Fc IgG binding activity and shows that the mutant (Ro52-Δ2-D355A) is defective in this binding activity.

**Figure 5:**
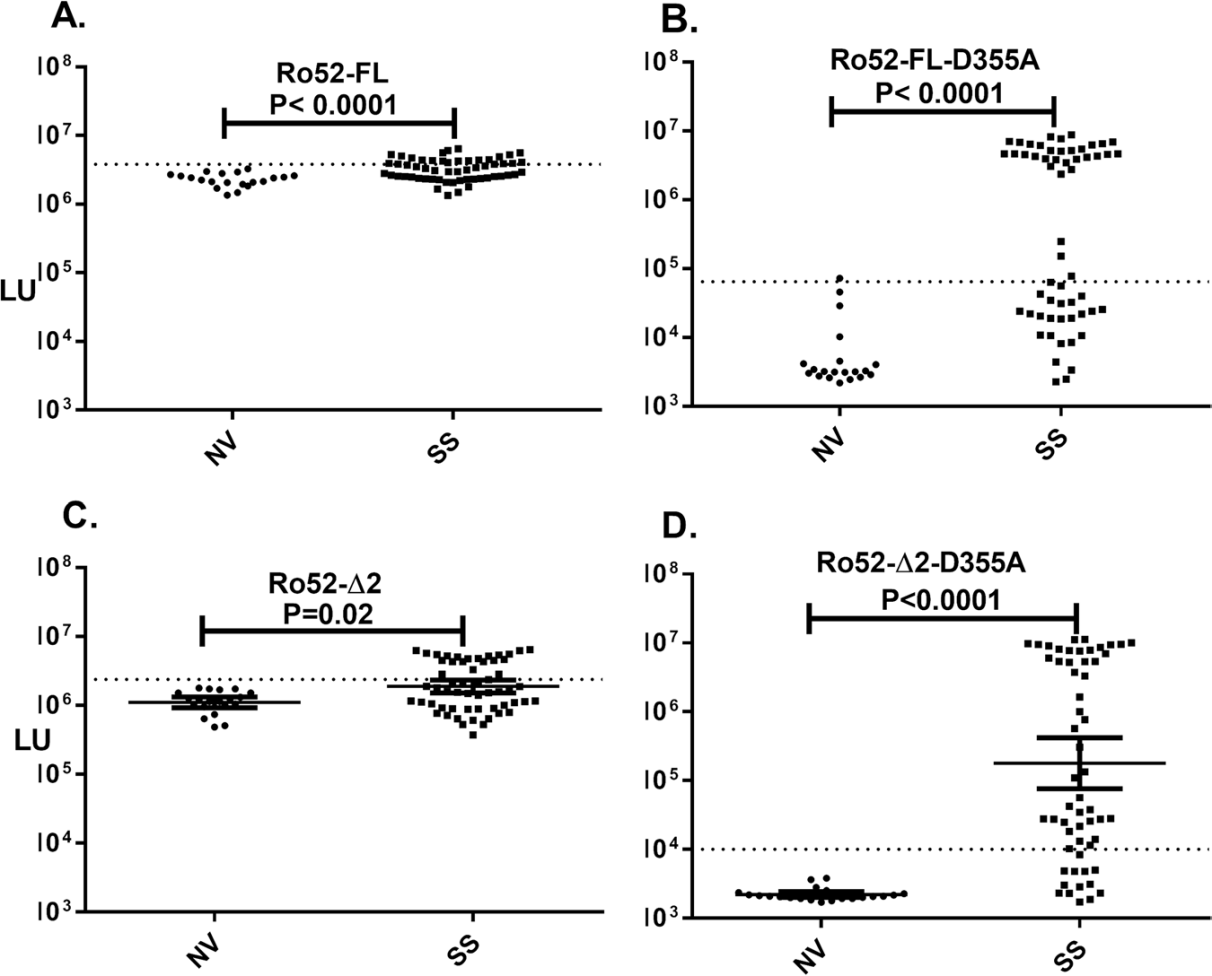
Robust autoantibodies are directed against the C-terminus of Ro52 in SS. Autoantibodies were examined in the cohort against **(A)** full-length wild type Ro52, (**B**) a full-length Ro52 mutant (Ro52-D355A), (**C**) the wild type C-terminus of Ro52 (Ro52-Δ2), and (**D**) a Ro52 C-terminal mutant (Ro52-Δ2-D355A). Each dot represents an individual subject from the cohort and the geometric mean of the antibody levels for normal volunteers (NV) and SS subjects is shown by the colored horizontal bar. The dotted lines for each antigen represent the cut-off values for determining seropositivity. *P* values were calculated using the Mann-Whitney *U* test.

Based on the Ro52-Δ2-D335A mutant’s inability to bind immunoglobulins, we investigated whether SS patients harbored autoantibodies directed against this region. As previously reported^33^, LIPS testing of full-length wild type Ro52 protein with 1 μl of undiluted sera revealed very high non-specific binding, yet statistically significant higher autoantibody levels in the SS subjects compared to the healthy volunteers (Fig. 5A). To address the contribution of Ro52’s immunoglobulin-binding domain to this activity, the full-length Ro52-D335A mutant, defective in immunoglobulin binding activity was employed. This mutant protein lost its antibody pull-down activity in tests with 1 μl of undiluted healthy control sera, but exhibited highly robust immunoreactivity with the SS serum samples (Fig. 5B). Calculation of the diagnostic utility of Ro52-D335A showed it had 58% sensitivity and 95% specificity, which matched the diagnostic performance of the N-terminal fragment. To formally determine whether autoantibodies are directed against Ro52’s C-terminal immunoglobulin-binding domain, the C-terminal fragment (Ro52-Δ2) and the mutant of this region defective in immunoglobulin binding (Ro52-Δ2-D355A) were tested. Consistent with our previous serological findings^35^, the wild C-terminal Ro52 protein fragment had a high background with slightly higher levels of immunoprecipitation in SS subjects than healthy volunteers (Fig. 5C). In contrast, the C-terminal mutant, Ro52-Δ2-D355A protein, showed a markedly different profile (Fig. 5D). In healthy volunteer sera, this mutant protein fragment showed negligible binding activity with a GML of 2201 LU (Fig. 5D). However, in SS subjects, this mutant protein revealed previously hidden robust autoantibody binding with a GML of 178,400 LU (95% CI: 75,820–419,800). Further comparison by Spearman Rank correlation of the antibody levels directed against the Ro52-Δ2-D355A mutant and N-terminal fragment of Ro52 (Ro52-Δ1) showed relative concordance (*R* = 0.78; *p* < 0.0001) in immunoreactivity. Together these results highlight the fact that autoantibodies are directed against both the N- and C-terminus of Ro52.

### Analysis of autoantibodies against the N-and C-terminal sub-fragments of Ro52

**Figure 6:**
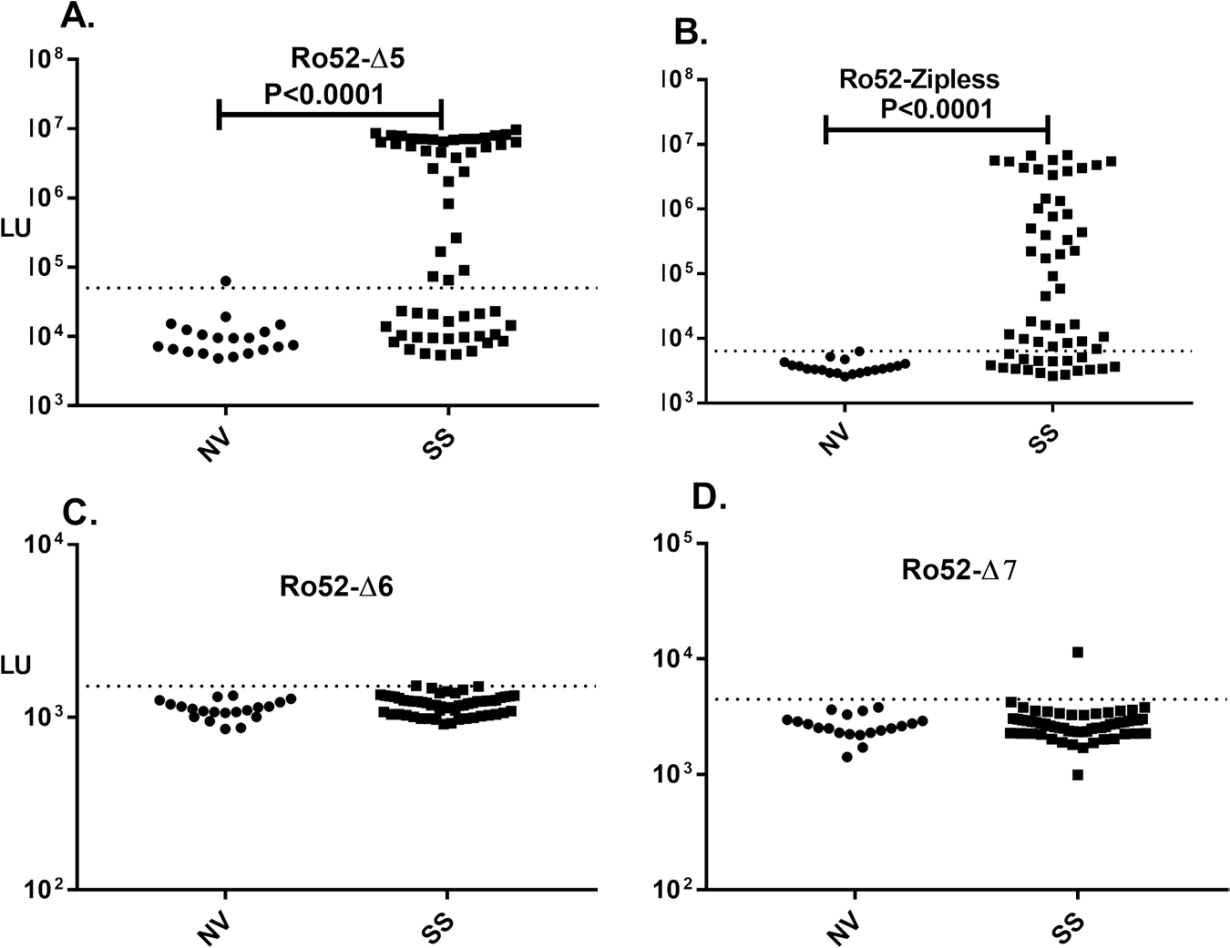
C-terminal deletion mutants eliminate the detection of conformational autoantibodies directed against this region. Evaluation of autoantibodies are shown to different N-terminal and C-terminal Ro52 deletion mutants in cohort. Ro52 deletion mutants included: (**A**) Ro52-Δ5; an N-terminal Ro52 mutant of 140 amino acids, (**B**) Ro52-Δ1-zipless; an internal deletion mutant eliminating the leucine zipper in the N-terminus of Ro52 (**C**) Ro52-Δ6; a C-terminal encompassing the first half of the immunoglobulin-binding region and (**D**) Ro52-Δ7; a C-terminal deletion mutant encompassing the second half of the immunoglobulin-binding region.

LIPS was previously used to successfully map antigenic regions in the N-terminus of Ro52 containing protein fragments of 50–140 amino acid residues^35^. Here we further attempted to more finely map the antigenic epitopes within the N- and C-terminus of Ro52. First, we retested an N-terminal Ro52-∆5 protein fragment of 140 amino acids (spanning amino acids 129–273) in the current cohort and found it had 58% sensitivity and 100% specificity for the diagnosis of SS (Fig. 6A). The high levels of autoantibody levels observed with this fragment in SS compared to the normal volunteers was highly statistically significant (Mann Whitney U test; p < 0.0001). Based on the report that the leucine zipper within the N-terminus might represent a potential immunodominant epitope^30^, a mutant (Ro52-zipless) bearing an internal deletion removing the leucine zipper region was tested. Interestingly, this Ro52 fragment devoid of the leucine zipper still retained high levels of immunoreactivity and showed 56% sensitivity (Fig. 6B), which was also highly significant (p < 0.001). To more accurately map epitopes in the C-terminus of Ro52, we generated two protein fragments, Ro52-∆6 and Ro52-∆7, spanning the immunoglobulin binding region. However, LIPS testing of Ro52-Δ6 (Fig. 6C) and Ro52-∆7 revealed a low background with no significant seropositivity in any of the SS samples (Fig. 6D). Taken together, these results suggest that the N-terminus of Ro52 contains both linear and conformational epitopes, but the C-terminal immunoglobulin-binding domain of Ro52 is mainly targeted by autoantibodies requiring the proper folding of this entire region.

### Rheumatoid factor, which are autoantibodies against the Fc region of IgG, show overlap with Ro52 seropositivity

**Figure 7:**
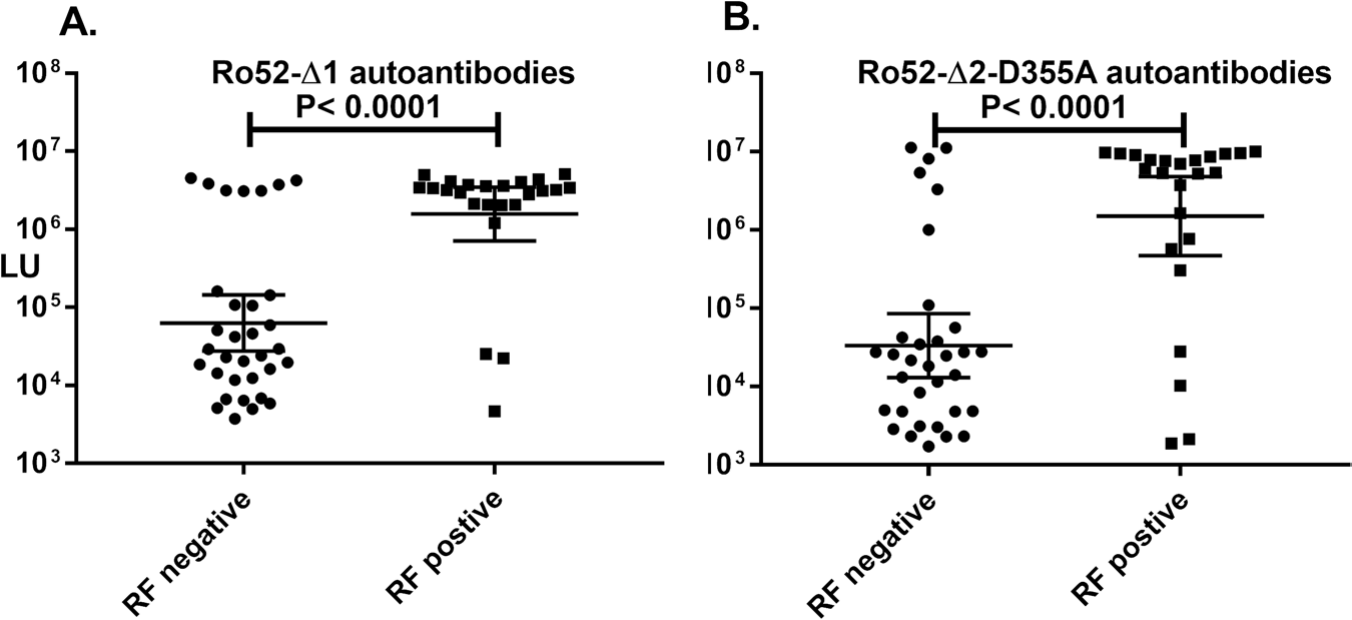
Rheumatoid factor autoantibodies co-segregate with Ro52 autoantibodies. SS subjects were segregated by their RF status (positive or negative) and the autoantibody levels against the (**A**) Ro52-Δ1 and (**B**) Ro52-Δ2-D355A protein fragments were then plotted in the subjects from each group. Each dot represents an individual SS subject and the geometric mean of the antibody levels in each group is shown by the horizontal bar. The *P* value was calculated using the Mann-Whitney *U* test.

Based on the finding that the C-terminal immunoglobulin binding region of Ro52 was an autoantibody target, we speculated that Ro52’s role in ADIN, the intracellular destruction of antibody-coated pathogens, might be related to its autoantigenicity. Interestingly, another highly prevalent autoantibody target in SS, rheumatoid factor (RF), binds the Fc region of IgG^45,46^. Therefore, we hypothesized that the loss of tolerance and development of autoantibodies against both Ro52 and the Fc region of immunoglobulins might mechanistically occur together due to ADIN, in which both proteins are associated with a pathogen-protein complex^17^. To gain additional evidence for this relationship, the seropositivity of RF in the SS subjects was examined and compared to autoantibody against the N- and C-terminal of Ro52. Seropositivity against the N-terminal fragment of Ro52 (Ro52-Δ1) in SS was found to highly segregate (Mann Whitney *U* test p < 0.0001) with RF seropositivity, in which the RF positive SS subjects had a GML of N-terminal Ro52 autoantibodies of 1,572,000 LU (95% CI: 711,100–3,473,000) compared to the RF negative samples of 63,089 LU (95% CI: 27,470–144,900) (Fig. 7A). Using the Fischer’s exact probability statistic, the SS subjects with Ro52 autoantibodies were highly likely to harbor RF (odds ratio 14.0, 95% CI: 3.4–49.3). Autoantibodies against the C-terminal Ro52 mutant (Ro52-Δ2-D355A) showed a very similar profile to the N-terminus of Ro52 and had strong association (p < 0.0001) with RF (Fig. 7B). For additional insight, we also examined Ro60 and La autoantibodies in the cohort and found that these autoantibodies were also highly diagnostic of SS, in which the seropositivity overlapped with Ro52 and rheumatoid factor (Supplemental Fig. 3 and data not shown). These data highlight that autoantibodies for RF and Ro52 are commonly found together in SS.

## Discussion

Understanding why proteins are autoantigens in specific autoimmune diseases may reveal mechanisms of pathogenesis. Here, we explored Ro52’s autoantigenicity in SS. We first considered whether Ro52 co-regulated gene products might be novel autoantigens. However, most of these co-regulated molecules, including proteins involved in proteosomal degradation and cell signaling, were not autoantigens in SS patients. We also tested structurally-related TRIM molecules for their autoantigenicity and found little evidence that other TRIM molecules are independent autoantigens in SS. However, one TRIM member, TRIM38, a Ro52-coregulated gene showed autoantibody responses in SS, but these TRIM38 autoantibodies occurred only in Ro52 positive subjects. Consistent with a recent report^47^, additional competition experiments revealed that TRIM38 was a minor autoantigen in SS with both autoantibodies specific for TRIM38, as well as autoantibodies cross-reacting with shared epitopes of Ro52. The potential finding that TRIM38 is often co-expressed with Ro52 may favor epitope spreading of autoantibodies from Ro52 to similar epitopes in TRIM38.

Ro52 is the only TRIM protein known to function in ADIN for destroying immunoglobulin-pathogen complexes within cells^40^. Despite the critical role of Ro52’s C-terminus in binding immunoglobulins, multiple published studies have failed to detect autoantibodies against this region using bacterial recombinant Ro52 proteins with various solid-phase immunoassays including ELISA, reduced ELISA, and/or Western blotting^30,48,49^. In contrast, our previous investigations with LIPS detected statistically significant increased prevalence of autoantibodies in SS compared to the controls against this C-terminal half of Ro52 protein, albeit with a high assay background^33,35^. In the current study, we replicated those findings and further explored in detail the biological properties and immunoreactivity of the C-terminal region. First, using an equivalent wild type C-terminal fragment of murine Ro52, containing only limited contiguous amino acid stretches with the human ortholog, autoantibodies were detected in 23% of the subjects with SS. Second, using an *in vitro* assay, a recombinant C-terminal Ro52 protein was found to bind immunoglobulins. Moreover, a previously described single amino acid mutant (D335A) known to be defective in immunoglobulin binding and predicted to block an important interaction with a histidine hot-spot residue needed for Fc binding^28^, was generated. Our studies with this mutant showed it disrupted immunoglobulin pull-down activity, but retained its antigenic epitopes revealing robust autoantibody immunoreactivity in 65% of the SS subjects. The immunoreactivity against this C-terminal protein fragment also strongly correlated (*R* = 0.78) with the level of autoantibodies against the N-terminus. Overall these findings suggest that this single amino acid mutant is sufficient to disrupt the relatively low affinity interactions between immunoglobulins and Ro52, but is insufficient to alter the overall structure of this region thereby allowing the detection of high affinity autoantibodies. Further analysis using two protein sub-fragments within the Ro52 C-terminus failed to detect autoantibodies. The likely explanation for the lack of immunoreactivity against these protein sub-fragments is that they are unable to fold properly and/or recapitulate the conformational epitopes associated with the complex tertiary structure of this region^28^.

**Figure 8:**
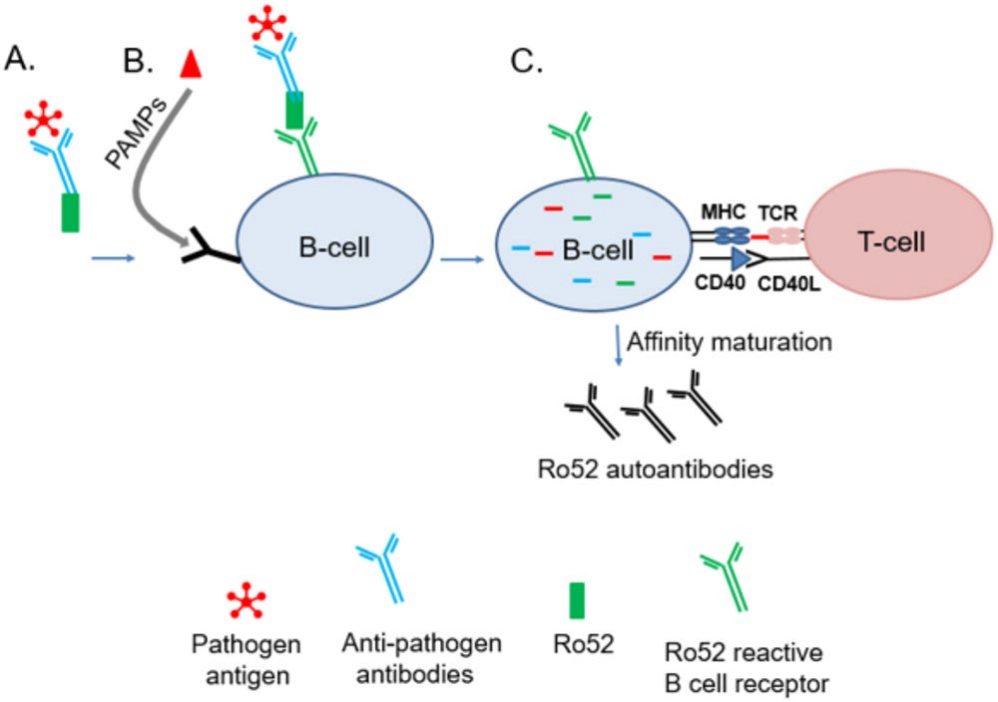
Model for the role of ADIN in anti-Ro52 autoantibody production. Ro52 is normally involved in antibody dependent intracellular neutralization (ADIN) of pathogens. (**A**) During infection, Ro52-bound antibody-coated pathogen complexes are released from dying cells. (**B**) In SS, autoreactive B-cell against Ro52 may bind and abnormally process the extracellular Ro52 along with the anti-pathogen antibody-antigen complex. The B-cell also receives co-stimulatory signals from pathogen–associated molecular pattern molecules (PAMPs). (**C**) Even though Ro52 was bound by the B-cell receptor, pathogen antigen is internalized, processed and presented by the B-cell. MHCII presentation of the pathogen antigen by the B-cell provides T-cell help fostered by co-stimulatory signals from B-T-cell interactions resulting in affinity maturation of the anti-Ro52 B-cells, which now produce high titer autoantibodies.

The findings that autoantibodies target the entire Ro52 molecule, including the N-terminal ubiquitin ligase and C-terminal immunoglobulin-binding region, link the process of Ro52 intracellular immunoglobulin-coated pathogen targeting to the proteosome with the protein’s autoantigenicity in SS and other rheumatological diseases. It was previously known that RF is highly prevalent in SS^45^ and both Ro52 autoantibodies and RF are present twenty years before the clinical diagnosis of SS^46^. We now present evidence that RF and autoantibodies against the N- and C-terminus of Ro52 often occur together in SS. Importantly, these findings highlight the remarkable convergence of autoantibodies targeting the Fc portion of IgG and the Fc-binding region of Ro52. We speculate that the autoantibody response against Ro52 in autoimmune conditions likely reflects some abnormality in immune signaling and/or pathogen clearance. We also propose a potential model (Fig. 8) for the generation of autoantibodies against Ro52 and the Fc region of Immunoglobulins, which relies on the “foreignness” of associated multivalent pathogen protein complexes containing Ro52 based on its normal function in ADIN. In this scenario, an ongoing infection releases large amounts of intracellular Ro52-bound to antibody coated-pathogen complexes from dying cells. The extracellular Ro52-antibody pathogen multivalent complexes are then co-captured by the B-cell receptor of autoreactive B-cells. Evidence for a similar co-capture model of a pathogen associated with a membrane bound autoantigen has recently been found^50^. The key feature of our model is that multivalent Ro52-immunoglobulin complexes associated with pathogen components are co-processed by antigen-presenting cells and thereby receive T-cell help from pathogen signals, resulting in robust Ro52 autoantibody production (Fig. 8). In SS, subtle defects in potentially any number of pathways including immune response, antigen processing and/or the trafficking of Ro52 peptides in antigen-presenting cells, may allow for enhanced pathogen-complex presentation on the cell surface. Similar models evoking pathogen-related T-cell help have been proposed for autoantibody production against RF^51^ and more recently for autoantibodies directed against the hinge region of immunoglobulins^52^. The potential role of abnormal antibody responses in the clinical features of SS is also supported by genome-wide association studies showing that some of the most highly disease-associated genes are involved in B-cell function (e.g. BTK) and antigen presentation (e.g. MHC)^53,54^. Overall, this model provides a mechanism in SS whereby immune responses against the pathogen are driving the loss of tolerance and resulting in the production of autoantibodies against Ro52 and the Fc region of IgG.

## Material and Methods

### Subject samples

In accordance with the human experimentation guidelines of the Department of Health and Human Services, sera were obtained from healthy volunteers (n = 20) and patients (n = 57) from the SS clinic, NIDCR, NIH, Bethesda, Maryland under an NIH institutional review board-approved protocol (IRB-D-0172). Informed written consent was provided by each participant. The subjects with SS fulfilled the revised American-European consensus criteria for Sjögren’s syndrome diagnosis^55^. The SS subjects used in the study were almost exclusively female with an average age of approximately 61 years (Supplemental Table 3). Rheumatoid factor (RF), autoantibodies against IgG, were determined in all the SS sera samples by ELISA in the Laboratory of Clinical Medicine, Clinical Center, NIH and this clinical data was revealed after Ro52 seropositivity was established by LIPS. Of note, RF clinical data was only available on five of the healthy controls and all were seronegative.

### Generation of Ro52-coregulated gene products and TRIM proteins for autoantibody testing

A publicly available database, Search-Based Exploration of Expression Compendium (SEEK; www.seek.princeton.edu)^39^, was mined to identify candidate Ro52 co-expressed genes. Querying with Ro52 (TRIM21) produced a list of the top 30 genes (Supplemental Table 1**)**. From this list, 12 proteins (PSMB9, PSMB8, PSME1, IRF-1, SAMD9L, DTX3L, IFI35, NMI, TRIM38 and TRIM22) were made as recombinant proteins for LIPS testing using the pREN2 vector^56^. Eight additional TRIM molecules were selected for autoantibody testing based on Ro52 homology and/or potential immune signaling relevance and were constructed in the pREN2 vector including TRIM5, TRIM13, TRIM15, TRIM31, TRIM39, TRIM43, TRIM56, and TRIM68. DNA sequence analysis was used to confirm the integrity of all constructs.

### Human and murine Ro52 proteins and mutants for autoantibody testing

Several previously described plasmids^33,35^, based on the pREN2 mammalian *Renilla* luciferase expression vector^56^, were used to generate recombinant Ro52 fusion proteins for LIPS testing, including full-length human Ro52 and three deletion mutants Ro52-Δ1 (spanning amino acid residues 2–273), Ro52-Δ2 (spanning amino acid residues 277–475) and Ro52-Δ5 (spanning amino acid residues 129–273). Additional constructs were made using custom primers, PCR and standard molecular techniques as previously described^56^. Two new pREN2 constructs for Ro52 contained protein fragments derived from the C-terminus of the protein were built including Ro52-Δ6 (spanning amino acid residues 277–339), and Ro52-Δ7 (spanning amino acid residues 349–475). Point mutants and internal deletion mutants of Ro52 were generated by the Q5 mutagenesis kit (New England Biolabs) with custom primers including Ro52-D355A, Ro52-Δ2-D355A¸and an internal deletion mutant (Ro52-Zipless) eliminating amino acids within the leucine zipper in the N-terminus of Ro52. The choice of the point mutant in the C-terminus of Ro52 was based on published crystallography and known immunoglobulin-binding activity of alanine mutants^28^. A schematic of the various Ro52 constructs used in this study are shown in Supplemental Fig. 4.

In addition to studying human Ro52, autoantibody responses against murine Ro52 were examined by LIPS using pREN2 constructs encompassing two different fragments spanning the first (mRo52-Δ1; amino acid residues 2–273) and second half of the molecule (mRo52-Δ2; amino acid residues 277–475). Lastly, autoantibodies were also evaluated using full-length La protein^33^ and a previously described protein fragment of Ro60^37^. DNA sequencing was used to validate the different plasmid constructs.

### *Renilla* luciferase fusion protein production

Using Fugene-6, Cos-1 cells were transfected with individual pREN2 expression vectors to produce recombinant light-emitting fusion proteins for the different protein or protein fragments^57^. Forty-eight hours later, crude lysates containing these recombinant proteins were harvested from Cos1 cells. This was performed by rinsing the Cos1 cells once with PBS before scraping and harvesting the cell layer in lysis buffer A (20 mM Tris, pH 7.5, 150 mM NaCl, 5 mM MgCl_2_,1% Triton X-100 containing protease inhibitors). The lysates were centrifuged twice at 13,000 × g. Supernatants were collected and used immediately or stored frozen. A tube luminometer (20/20 from Turner Scientific) was used with coelenterazine substrate mix (Promega, Madison, WI) to determine the luciferase activity of the lysate in LU.

### LIPS analysis

Using a 96-well plate format, all LIPS assays were performed at room temperature as described^57^. Serum samples were first diluted 1:10 in assay buffer A (20 mM Tris, pH 7.5, 150 mM NaCl, 5 mM MgCl_2_, 1% Triton X-100) in 96-well polypropylene microtiter plates. To quantify antibody titers by LIPS, 40 μl of buffer A, 10 μl of diluted human plasma (1 μl equivalent), and 50 μl Ruc-antigen Cos1 cell extract diluted in buffer A (typically 1 × 10^7^ LU), were added to each well and incubated for 1 hour at room temperature. Next, 7 μl of a 30% suspension of Ultralink protein A/G beads (Pierce Biotechnology, Rockford, IL) in PBS were added to the bottom of each well of a 96-well filter HTS plate (Millipore, Bedford, MA). The 100 μl antigen-antibody reaction mixture was then transferred to filter plates and incubated for 1 hour at room temperature on a rotary shaker. Antibody complexes bound to the protein A/G beads were washed 10 times with buffer A and twice with PBS. After the final wash, LU were measured in a Berthold LB 960 Centro microplate luminometer (Berthold Technologies, Bad Wilbad, Germany) using coelenterazine substrate mix (Promega, Madison,WI).

Additional competition experiments were used to characterize the autoantibodies in six SS subjects co-positive for Ro52 and TRIM38 autoantibodies (Supplemental Table 2). Competition experiments were performed essentially as described using LIPS tube assay^56^. Sera was diluted 1:300 in buffer A and incubated with either 80 μl of control extract or Myc-tagged Ro52 extract as competitor for 30 min before adding either the *Renilla* luciferase-Ro52 fusion extract or *Renilla* luciferase-TRIM38 extract (1 million LU input). After an additional 30 min, protein A/G beads were added and the samples were processed. LU were then measured in a tube luminometer. Background LU (beads plus extract but no sera) were subtracted before calculating the percent autoantibody activity remaining.

### *In vitro* protein-protein interaction studies of Ro52 with IgG1

Interactions between IgG1 and Ro52 were studied using a previously described method based on luciferase tagged proteins to examine protein-protein interactions^42^. The detector employed was a recombinant protein fragment derived from the Fc region of IgG1 (amino acids 233–449) generated as a fusion protein with *Renilla* luciferase from the pREN2-IgG-Fc plasmid. As a negative control, recombinant protein produced from pREN2-IgG-Fc-mut, an internal deletion mutant within the Fc region of IgG1 missing critical amino acids residues (His435, Asn436 and His437) required for interactions with protein A, protein G and Ro52^25,58^, was also generated. The corresponding binding targets, the C-terminal Ro52 protein (Ro52-Δ2) and mutant protein fragment (Ro52-Δ2-D355A), were generated as a chimeric fusion protein with glutathione-S-transferase. To build these constructs, a new vector derived from pCAF2, called cGST, involved subcloning the cDNA for GST within the polylinker of this mammalian expression vector. This cGST vector was then used to subsequently subclone the C-terminal DNA fragment of Ro52 (cGST-Ro52-Δ2) and the C-terminal Ro52 mutant (cGST-Ro52-Δ2-D355A). DNA sequence analysis was used to confirm the integrity of all constructs. These plasmid constructs were then expressed in Cos1 cell and lysates containing recombinant protein were obtained as described above.

To examine protein-protein interactions, buffer A, cell lysate containing equal amounts of *Renilla* luciferase-Fc IgG1 fusion protein or *Renilla* luciferase-mutant Fc IgG1 activity (1 × 10^7^ LU) were mixed with cell extract in 1.5 ml microfuge tubes. Extract (30 μl) containing either recombinant GST-Ro52-Δ2 or GST-Ro52-Δ2-mutant protein was then added. Lastly, either glutathione beads (10 μl packed beads) or protein A/G beads (5 μl) were added and the whole mixture incubated at 4 °C with tumbling for 1 hour. The tubes were briefly centrifuged to pellet the beads and washed three times with 1.5 ml of buffer A and then once with 1 ml of PBS. Following the last wash, the amount of luciferase-antigen bound was determined by measuring luminescence with coelenterazine substrate as described using the 202/20 Turner tube luminometer^42^.

### Statistical analysis

GraphPad Prism software (San Diego, CA) was used for generating the scatter plots and statistical analyses. Results for quantitative antibody levels were reported as the geometric mean levels (GML) ± the 95% confidence interval (CI). As shown in the scatter plot figures, Mann-Whitney *U* tests were used for comparison of the antibody levels between healthy volunteers and SS subjects with a significance level of *P* < 0.05. For the calculation of sensitivity and specificity, a statistically based cut-off value for each antigen was derived from the mean of the control samples plus three standard deviations. The Fischer’s exact test was used to assess the statistical significance of the differences in autoantibody prevalence between the SS subjects and healthy volunteers and/or for calculating the odds ratio. Correlations between antibody responses against protein fragments were assessed by the Spearman rank correlation coefficient.

Heatmap analysis was employed to visualize anti-Ro52 and TRIM autoantibody responses. The healthy volunteers served as the reference group. To eliminate any false positives in the healthy controls, a more stringent cut-off value for seropositivity required subjects to at least have autoantibody levels above the mean plus 5 standard deviations of the healthy controls. For each subject, antibody levels were then calculated as a *Z* score value and then color-coded to reflect the relative antibody levels above the controls as described^37^. SS subjects seropositive for at least one TRIM protein are shown.

## Electronic supplementary material

Supplementary Information
