# Supplementary material for: Autoantibodies against the Immunoglobulin-Binding Region of Ro52 Link its Autoantigenicity with Pathogen Neutralization
Source: Sci Rep. 2018 Feb 20;8:3345. doi: 10.1038/s41598-018-21522-7 (PMC5820281; doi:10.1038/s41598-018-21522-7)
Supplement: Supplementary file 1 — Supplementary Information [file 41598_2018_21522_MOESM1_ESM.pdf]

Autoantibodies against the Immunoglobulin-Binding Region of Ro52 Link its Autoantigenicity with Pathogen Neutralization

Peter D. Burbelo, Leyla Y. Teos, Jesse L. Herche, Michael J. Iadarola and Ilias Alevizos

Legends:

Supplemental Figure 1: LIPS analysis of autoantibodies against other TRIM proteins.
Autoantibody levels against additional TRIM proteins including **(a)** TRIM5, **(b)** TRIM13, **(c)** TRIM15, **(d)** TRIM31, **(e)** TRIM39, and **(f)** TRIM68 were examined in the SS cohort by LIPS. Each dot represents an individual subject and the geometric mean of the antibody levels in control and SS group is shown by the horizontal bar. The dotted line is the cutoff value for each antigen based on the control group. *P* values were calculated using the Mann-Whitney *U* test.

Supplemental Fig. 2: Alignment of human and murine Ro52 orthologs.
The amino acid sequences for human and murine Ro52 proteins show minimal contiguous homology. Similar amino resides shared by both proteins are denoted by the plus sign. Note the lack of contiguous identical amino acids stretches greater than seven amino acid residues.

Supplemental Fig.3: LIPS analysis of autoantibodies Ro60 and La.
Autoantibody levels against Ro60 and La were examined in the SS cohort by LIPS. Each dot represents an individual subject and the geometric mean of the antibody levels in control and SS group is shown by the horizontal bar. *P* values were calculated using the Mann-Whitney *U* test.

Supplemental Fig. 4: Schematic of the different Ro52 deletion and point mutants used in this study.
The human Ro52 protein is composed of 475 amino acid residues. The N-terminus of Ro52 contains the ubiquitin ligase domain and leucine zipper region. The C-terminus contains the immunoglobulin-binding region.

Suppl. Table 1 Identification of Ro52-Coregulated Genes by SEEK analysis

Suppl. Table 2 Characterization of TRIM38 immunoreactivity in Ro52-TRIM38 Copositive Sera

Suppl. Table 3. Demographics of the Cohort of Normal Volunteers and subjects with SS

Supplemental Fig. 1

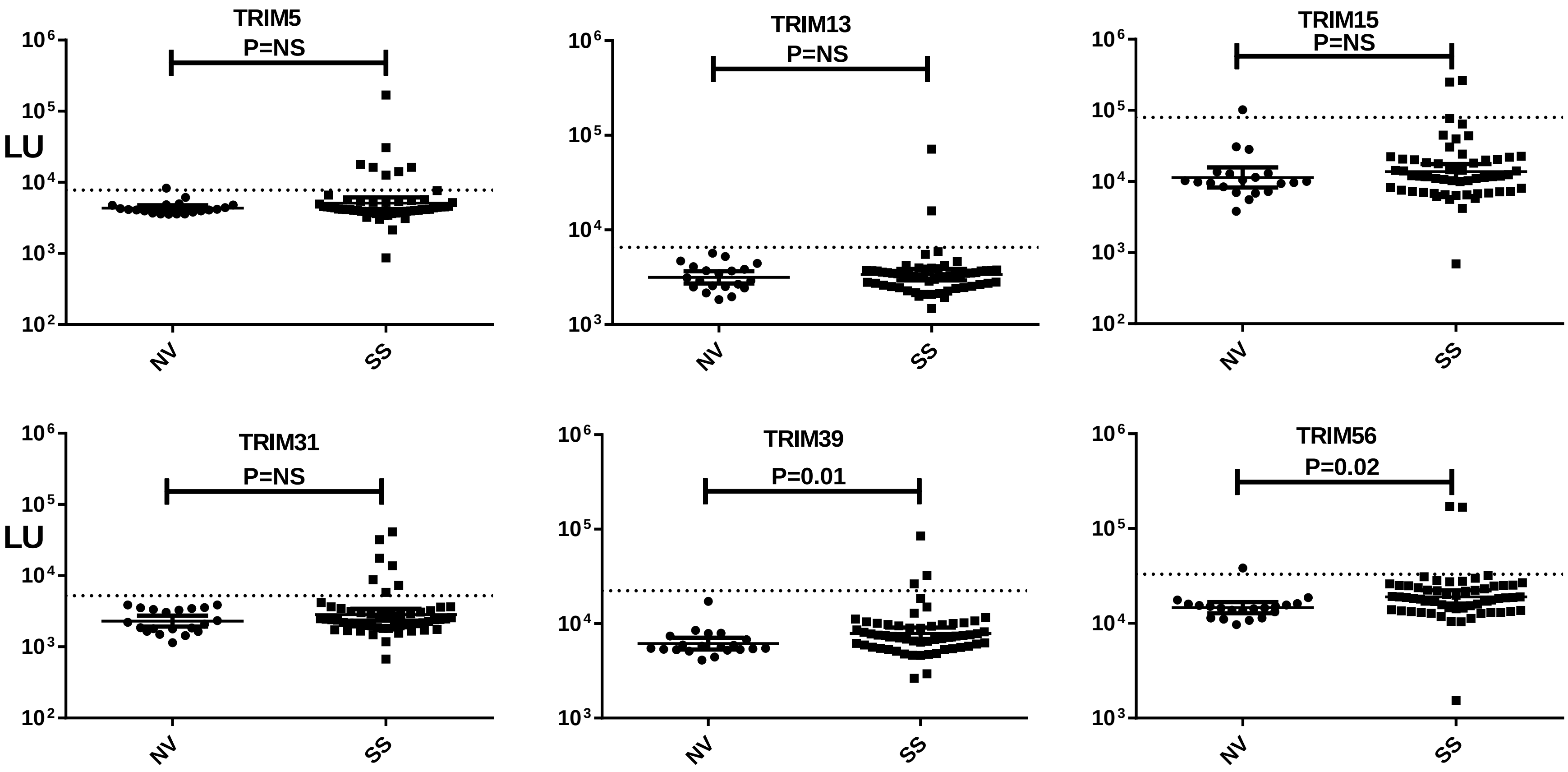

Supplemental Fig. 2

```
MASAARLTMMWEEVTCPICLDPFVEPVSIECGHSFCQECISQVGKGGGSVCPVCRQRFLL   60 (H)
M     L  MWEEVTC ICLDP VEP+SIECGH FC+ECI +VGK GGS CP CRQ+FLL
MS----LEKMWEEVTCSICLDPMVEPMSIECGHCFCKECIFEVGKNGGSSCPECRQQFLL   56 (M)

KNLRPNRQLANMVNNLKEISQEAREGTQGERCAVHGERLHLFCEKDGKALCWVCAQSRKH  120 (H)
+NLRPNR +ANMV NLK+I+Q  ++ TQ   C  HGE+LHLFCE+DG+ALCWVCAQS KH
RNLRPNRHIANMVENLKQIAQNTKKSTQETHCMKHGEKLHLFCEEDGQALCWVCAQSGKH  116 (M)

RDHAMVPLEEAAQEYQEKLQVALGELRRKQELAEKLEVEIAIKRADWKKTVETQKSRIHA  180 (H)
RDH  VP+EEAA+ YQEK+ VAL +LR+ +ELAEK+E+++ ++R DWK+ ++TQKSRIHA
RDHTRVPIEEAAKVYQEKIHVALEKLRKGKELAEKMEMDLTMQRTDWKRNIDTQKSRIHA  176 (M)

EFVQQKNFLVEEEQRQLQELEKDEREQLRILGEKEAKLAQQSQALQELISELDRRCHSSA  240 (H)
EF  Q + L +EEQRQLQ LEKD+RE LR+LG+KEA+LA+++QALQELISEL+RR   S
EFALQNSLLAQEEQRQLQRLEKDQREYLRLLGKKEAELAEKNQALQELISELERRIRGSE  236 (M)

LELLQEVIIVLERSESWNLKDLDITSPELRSVCHVPGLKKMLRTCAVHITLDPDTANPWL  300 (H)
LELLQE VI+LERS SWNL  LDI +P+L S C VPG KKMLRTC VHITLD +TAN WL
LELLQEVRIILERSGSWNLDTLDIDAPDLTSTCPVPGRKKMLRTCWVHITLDRNTANSWL  296 (M)

ILSEDRRQVRLGDTQQSIPGNEERFDSYPMVLGAQHFHSGKHYWEVDVTGKEAWDLGVCR  360 (H)
I+S+DRRQVR+GDT Q++  NEERF +YPMVLGAQ F SGK YWEVDVT KEAWDLGVCR
IISKDRRQVRMGDTHQNVSDNEERFSNYPMVLGAQRFSSGKMYWEVDVTQKEAWDLGVCR  356 (M)

DSVRRKGHFLLSSKSGFWTIWLWNKQKYEAGTYPQTPLHLQVPPCQVGIFLDYEAGMVSF  420 (H)
DSV+RKG F LS ++GFWTIWLW +  YEAGT PQT LH+QVPPCQ+GIF+DYEAG+VSF
DSVQRKGQFSLSPENGFWTIWLW-QDSYEAGTSPQTTLHIQVPPCQIGIFVDYEAGVVSF  416 (M)

YNITDHGSLIYSFSECAFTGPLRPFFSPGFNDGGKNTAPLTLCPLNIGSQGSTDY*  475(H)
YNITDHGSLIY+FSEC F GPLRPFF+ GFN  G N APL LCPL +
YNITDH GSLIYTFSECVFAGPLRPFFNVGFNYSGGNAAPLKLCPLKM* 462 (M)
```

Supplemental Fig. 3

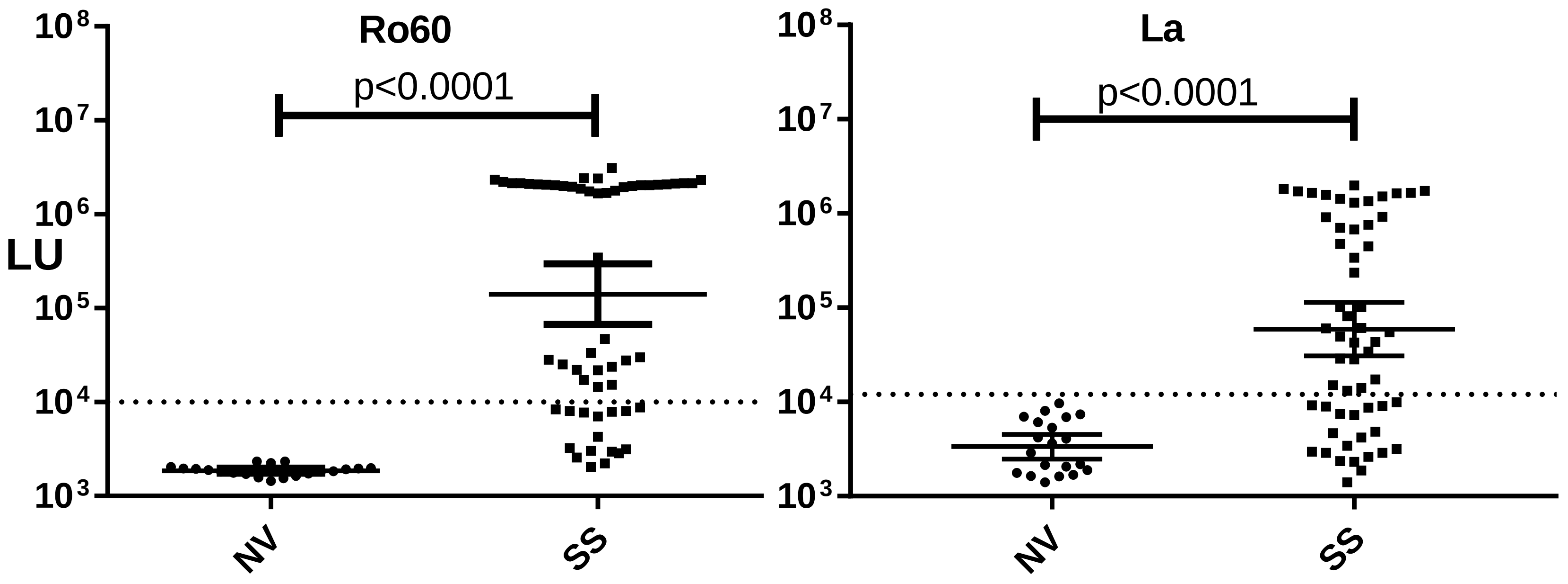

Supplemental Fig. 4

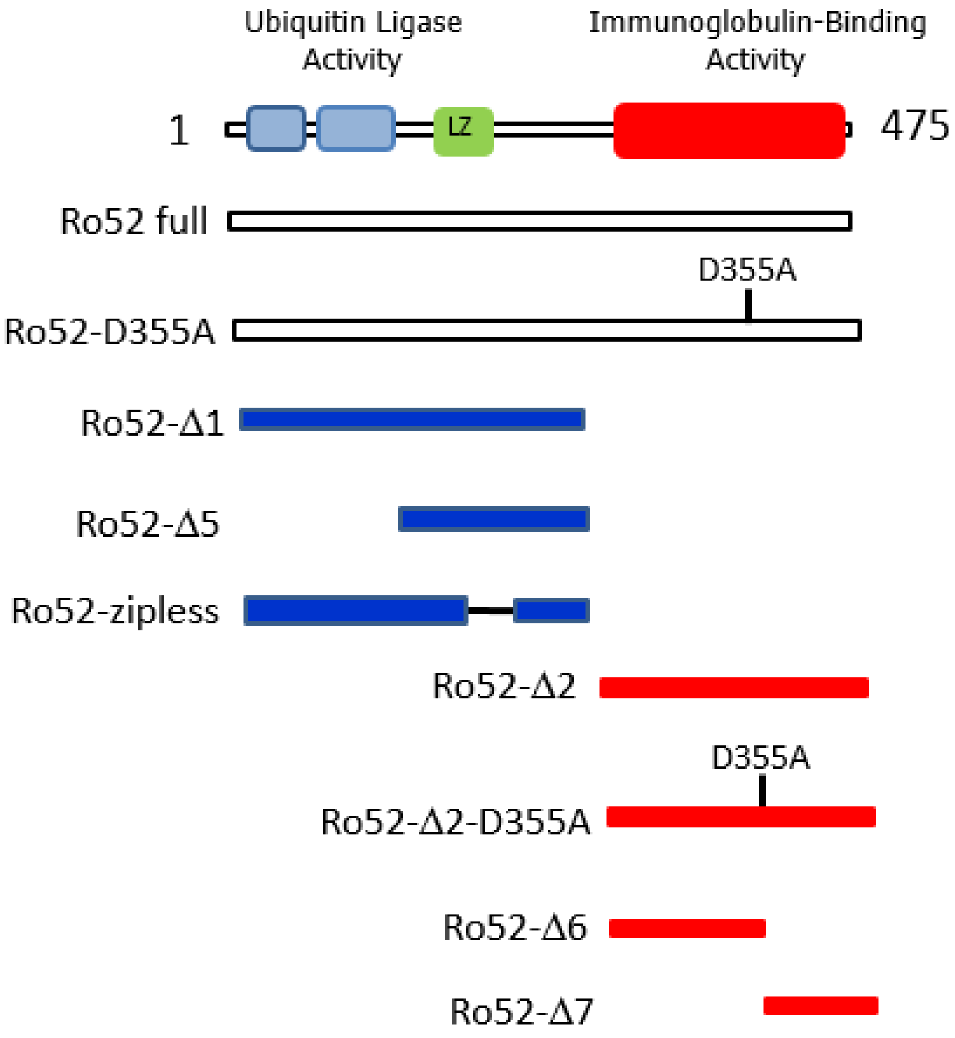

Supplemental Table 1

Gene	Rank by SEEK Analysis	SEEK Score
IRF1	1	3.02
PSMB9	2	2.95
PSMB8	3	2.91
SAMD9L	4	2.91
PARP14	5	2.86
TAP2	6	2.77
DTX3L	7	2.77
UBA7	8	2.77
SP100	9	2.76
IFI35	10	2.74
NLRC5	11	2.73
ZNFX1	12	2.73
PLSCR1	13	2.69
NMI	14	2.65
PSMB10	15	2.64
TRIM38	16	2.64
TAP1	17	2.64
DDX60L	18	2.63
MLKL	19	2.62
CTSS	20	2.62
SP110	21	2.60
TAPBP	22	2.60
PARP9	23	2.59
HLA-E	24	2.59
TRIM22	25	2.56
RTP4	26	2.54
STAT6	27	2.54
PSME1	28	2.53
RNF213	29	2.52
PML	30	2.52

Supplemental Table 2

Serum sample	Ro52 Competition With Ruc-Ro52 (% remaining)	Ro52 Competition With Ruc-TRIM38 (% remaining)
41	0.08	0.44
47	0.18	0.30
30	0.06	0.41
44	0.04	0.33
45	0.00	0.54
48	0.01	0.28

Competition experiments were performed essentially as described using LIPS tube assay (Burbelo et al., 2005). Diluted sera (1:300) was incubated with either 80 μl of control extract or Myc-tagged Ro52 extract as competitors for 30 min before adding either the *Renilla* luciferase-Ro52 fusion extract or *Renilla* luciferase-TRIM38 extract (1 million LU input). After an additional 30 min, protein A/G beads were added and processed by the LIPS tube assay. Background light units (beads plus extract but no sera) were subtracted before calculating the percent autoantibody activity remaining. As shown, competitor Myc-Ro52 efficiently competed the Ruc-Ro52 signal, but only partially competed away the Ruc-TRIM38 signal in the serum samples.

Supplemental Table 3

		Healthy Volunteers N=20	Subjects with Sjögren's Syndrome N=57
Percent Females/males		80%/20%	98%/2%
Age, years (mean, ± SD)		47.1± 13.2	60.9 ± 12.1
Ethnicity	WHITE	10 (50%)	41 (72%)
	AFRICAN	8 (40%)	7 (13%)
	ASIAN	2 (10%)	6 (10%)
	UNSPECIFIED	0 (5.3%)	3 (5%)
